# Supplementary material for: A Critical View of the Application of the APEX Software (Aqueous Photochemistry of Environmentally-Occurring Xenobiotics) to Predict Photoreaction Kinetics in Surface Freshwaters
Source: Molecules. 2019 Dec 18;25(1):9. doi: 10.3390/molecules25010009 (PMC7017383; doi:10.3390/molecules25010009)
Supplement: Supplementary file 1 [file molecules-25-00009-s001.zip › 3.2.4_gcc-4.4.0.zip!/3.2.4_gcc-4.4.0/3.2.4_gcc-4.4.0/doc/octave/html/interpreter/Integer-Arithmetic.html]

Integer Arithmetic - Untitled


Up: Integer Data Types


---

#### 4.4.1 Integer Arithmetic

While many numerical computations can't be carried out in integers,
Octave does support basic operations like addition and multiplication
on integers. The operators `+`, `-`, `.*`, and `./`
work on integers of the same type. So, it is possible to add two 32 bit
integers, but not to add a 32 bit integer and a 16 bit integer.

The arithmetic operations on integers are performed by casting the
integer values to double precision values, performing the operation, and
then re-casting the values back to the original integer type. As the
double precision type of Octave is only capable of representing integers
with up to 53 bits of precision, it is not possible to perform
arithmetic with 64 bit integer types.

When doing integer arithmetic one should consider the possibility of
underflow and overflow. This happens when the result of the computation
can't be represented using the chosen integer type. As an example it is
not possible to represent the result of 10 - 20 when using
unsigned integers. Octave makes sure that the result of integer
computations is the integer that is closest to the true result. So, the
result of 10 - 20 when using unsigned integers is zero.

When doing integer division Octave will round the result to the nearest
integer. This is different from most programming languages, where the
result is often floored to the nearest integer. So, the result of
`int32(5)./int32(8)` is `1`.

— Function File: **idivide** (x, y, op)  
> Integer division with different round rules. The standard behavior of
> the an integer division such as a `./` b is to round
> the result to the nearest integer. This is not always the desired
> behavior and `idivide` permits integer element-by-element
> division to be performed with different treatment for the fractional
> part of the division as determined by the op flag. op is
> a string with one of the values:
>
> "fix": Calculate a `./` b with the fractional part rounded towards zero. "round": Calculate a `./` b with the fractional part rounded towards the nearest integer. "floor": Calculate a `./` b with the fractional part rounded downwards. "ceil": Calculate a `./` b with the fractional part rounded upwards.
>
> If op is not given it is assumed that it is `"fix"`.
> An example demonstrating these rounding rules is
>
> ```
>           idivide (int8 ([-3, 3]), int8 (4), "fix")
>           => int8 ([0, 0])
>           idivide (int8 ([-3, 3]), int8 (4), "round")
>           => int8 ([-1, 1])
>           idivide (int8 ([-3, 3]), int8 (4), "ceil")
>           => int8 ([0, 1])
>           idivide (int8 ([-3, 3]), int8 (4), "floor")
>           => int8 ([-1, 0])
> ```
>
>
>
>
> **See also:** ldivide, rdivide.
